# Pan-Am’s Farewell

**Published:** 1901-12

**Authors:** James B. Parke

**Affiliations:** Buffalo Evening News


**Pan-Am's Farewell.**

JAMES B. PARKE, JR., in *Buffalo Evening News*, November 2, 1901.

Good-bye,

It's the final call,

My last breath,

So come one, come all

And be in

At the death.

It will be the

Greatest sight to see—

All jollity,

Frivolity.

So come;

Be on hand as it will

Help some.

I know that as a

Financial proposition

My position

Is unique,

But as a show

I also know that no

One ever saw anything so

Beautiful.

So come and have a

Parting peek.

And after my

Demise

Be wise;

Just go and bury all your

Hammers and

Hatchets and

Knives and

Clubs and

Other implements,

For those instruments

Generally knock

The wrong people and

In my opinion

Your Uncle Sam's

Cool million

Won't shower much on any

Rough House

Cotillion.

Anyhow, don't forget that tomorrow

I'm a has-been,

So come in

Today

From everywhere about—

For tonight

My light

Goes

Out!

---

THE Infant Incubators installed at the Pan-American Exposition by the Qubata Company made a splendid record. Of the 52 infants brought to the institution 48 were delivered into the hands of their mothers at the close of the season in a healthy and prosperous condition. Four died in a few hours after arrival or were dead when they were brought in. This is a worthy record and it is interesting to note that the children's hospital in Bryant Street is to have an incubator ward, orders having been given to the Qubata company for the necessary apparatus. It will probably be a few weeks before the incubators are installed, as they have to be made in Germany and shipped thence to Buffalo.

---

THE appearance of smallpox in Buffalo may be considered as a legacy of the exposition. It was to be expected, when we consider the vast number of people that visit a city at such a time. Some will come unprotected from the disease and such are almost sure to bring it and spread it in their trail. The health commissioner succeeded in keeping the knowledge of its presence from the public, and in managing the condition so discreetly that no harm came to the exposition. The 12 or more cases that existed at that time recovered, and it was not until the close of the fair that the presence of the disease became generally known.

The disease is now increasing only moderately, but it may be expected to spread considerably more before it is stamped out. It took Chicago two years to rid itself of the contagion after the World's Fair, but that city was not prepared to deal with it as well as Buffalo is at the present time. The stringent measures adopted by the health department will bring it under control in a reasonable time; meanwhile, it is important for every person who has not been successfully vaccinated within a short time to submit to that preventive measure. There is no question in the minds of well informed persons as to its safety and reliability as a prophylactic. Revaccination is very important where the vaccine disease is not of recent date, and in all cases of liability to exposure.

